# Increased nerve twigs in small intestinal mucosa with programmed cell death-ligand 1 and somatostatin receptor type 2A expression in recurrent Crohn disease: A case report

**DOI:** 10.1097/MD.0000000000013492

**Published:** 2018-12-10

**Authors:** Maria Lucia Caruso, Federica Di Pinto, Antonia Ignazzi, Sergio Coletta, Anna Maria Valentini, Elisabetta Cavalcanti, Francesco De Michele

**Affiliations:** Department of Pathology, National Institute of Gastroenterology “S. de Bellis”, Research Hospital, Castellana Grotte, Bari 70013, Italy.

**Keywords:** Crohn disease, nerve, programmed cell death-ligand 1

## Abstract

**Rationale:**

Inflammatory bowel disease (IBD) patients manifest symptoms of disturbed gut function, such as neural sensory-motor changes. Programmed cell death-ligand 1 (PD-L1), normally present in neural tissue, exists in close apposition to the mucosal immune system and intestinal epithelium, and a bi-directional communication is known to occur at these interfaces. Somatostatin has been shown to suppress the inflammatory reaction, and has been used in several clinical trials to treat inflammatory disorders, such rheumatoid arthritis. Recently, somatostatin receptor type 2A, that regulates neurotransmission, proliferation, and apoptosis, has been recognized in IBD. Although prominent abnormalities in the morphology of the enteric nervous system have been observed in idiopathic IBD, they are more marked in Crohn disease.

**Patient concerns:**

A 55-year-old woman with recurrent Crohn disease, just surgically treated for ileal resection, have a stenotic complication.

**Interventions:**

At surgery 5 cm of preterminal ileum with stenosis and anastomotic ileocolic block was removed.

**Diagnoses:**

The histopathology showed a recurrent Crohn in fistulo-stenotic phase; the stenosis was mainly sustained by mass-forming, ganglioneuromatous hyperplasia. Normally very rare, fine nerve twigs extend up into mucosa but we found a new-formed fibrillary network, extending into the inflammation area at the subepithelial luminal site of the mucosa, that was positive to PD-L1 and somatostatin receptor type 2A (SSTR2A) immunostaining but not visualized in routinary stained slides.

**Outcomes:**

After surgery the patient was semestrally followed with clinical endoscopic evaluation that results uneventfully.

**Lessons:**

Our case shows that before surgery neuromatous abnormalities can be predicted by immunostained new-formed twigs in the mucosa.

## 1 Introduction

The “intestinal brain” comprises extrinsic and intrinsic innervations, the latter organized in 2 major plexuses, myenteric, and submucosal. Patients with inflammatory bowel disease (IBD) manifest symptoms of disturbed gut function, such as neural sensory-motor changes and altered secretion. Neurons of the enteric nervous system (ENS) also exist in close apposition to cells of the mucosal immune system and the intestinal epithelium, and bi-directional communication is known to occur at these interfaces.^[1,2]^ Specific connections between intestine and brain, the so-called intestine–brain axis, implemented by endocrine, neural and immune systems, are now being extensively investigated.^[3]^ Studying the role of programmed cell-death ligand 1 (PD-L1) in acute pain modulation, Chen revealed a mild inhibition of the first pain phase and an increased pain threshold in mice. PD-L1 has a great relevance in new oncological immunotherapy schemes, and is gaining importance in many other fields.^[4,5]^ Moreover, PD-L1, normally present in neural tissue, has been shown to suppress spinal cord synaptic transmission in the pain circuit, acting as a unique neuromodulator.^[6]^ Somatostatin has been shown to suppress the inflammatory reaction and somatostatin has been used in several clinical trials to treat inflammatory disorders, such as psoriasis and rheumatoid arthritis.^[7]^ Recently, somatostatin receptor type 2A (SSTR2A), that regulates neurotransmission, proliferation, and apoptosis,^[8]^ has been recognized in IBD.^[9]^ Although prominent abnormalities in the morphology of the ENS have been observed in idiopathic IBD, they are more marked in Crohn disease (CD) than in ulcerative colitis^[10]^ (UC) and in some studies are considered as hamartoma.^[11–13]^ Therefore, neuronal lesions have a true diagnostic role in CD, although it is debated whether the changes are merely secondary responses to chronic inflammation or have a direct pathogenic role.^[1,14]^ Structural changes in the gut ENS can include an increased number (hyperplasia) and/or increased size (hypertrophy) of nerve bundles and ganglion cells. The neural plexuses abnormalities are prevalently associated with the extent of the inflammatory infiltrate but are reported in both involved and non-involved CD areas.^[15,16]^ Moreover, the presence of plexitis has recently been shown to be predictive of CD recurrence^[17]^ and of an ominous disease evolution, suggesting that neuroprotection could decrease the disease severity. Well-oriented biopsies including muscolaris mucosae and a small portion of superficial submucosa are key material for a correct diagnosis of IBD. Ganglioneuronal involvement is well known as a diagnostic pattern of Crohn^[1]^ but like more commonly adopted diagnostic features such as transmural inflammation, aphthoid ulcers, fissuration, fistulas, lymphangiectasia, follicular hyperplasia, and fibrous stricturing, it is difficult to highlight in endoscopic grasp forceps biopsies. Mucosal suction biopsies, deep enough to include the submucosa plexus, are adequate to document nerve hypertrophy. However, it is difficult to visualize neurons and nerve bundles in conventionally stained haematoxylin and eosin sections, unless they are hypertrophic.^[14]^

## 2 Case presentation

**Figure 1:**
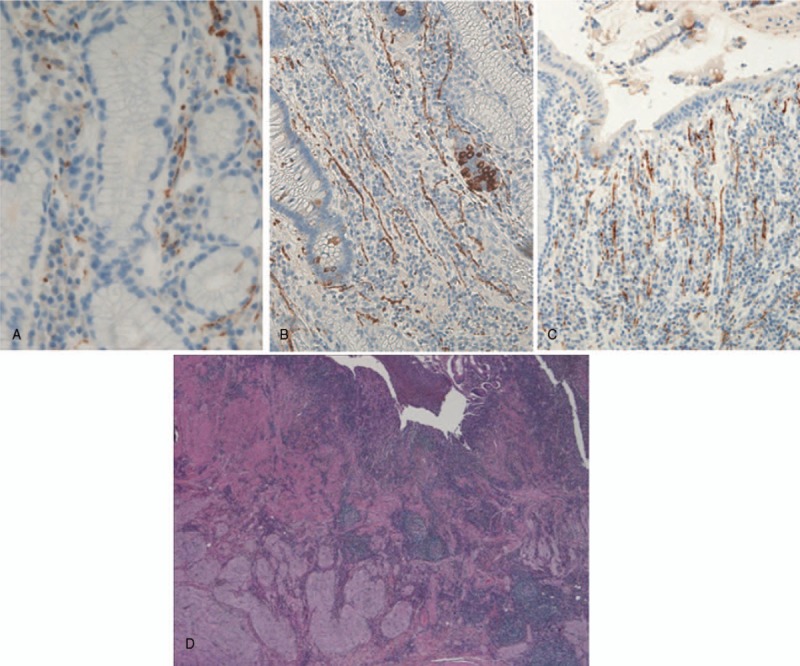
Nerve twigs running parallel to the crypts, synaptophysin immunostaining in: (A) rare twigs in control mucosa (cancer specimen) (400×); (B–C) numerous in Crohn (200×), predicting (D) ganglioneuromatous hyperplasia at the submucosa and muscolaris layer associated with marked follicular hyperplasia with pattern reminiscent of string of pearls, (HE, 20×).

**Figure 2:**
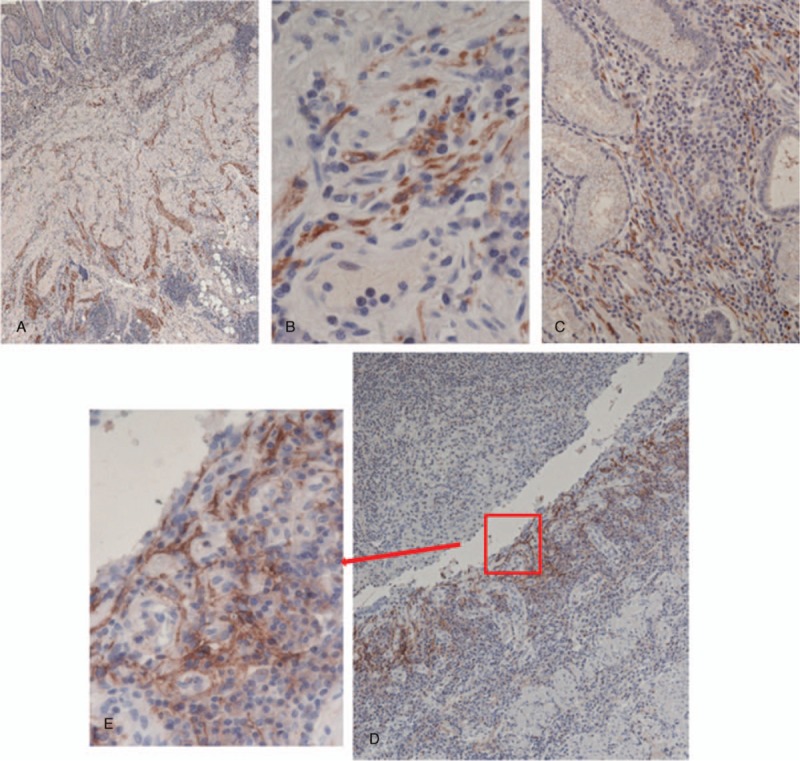
PD-L1 immunostaining in: (A–B) muscolaris mucosae, (20×–600×); (C) on heavy inflamed mucosa, (200×); (D–E) SSTR2A immunostaining in felt under luminal epithelium constituted by meshes of neuronal fragments, (100×–400×). PD-L1 = programmed cell death-ligand 1, SSTR2A = somatostatin receptor type 2A.

**Figure 3:**
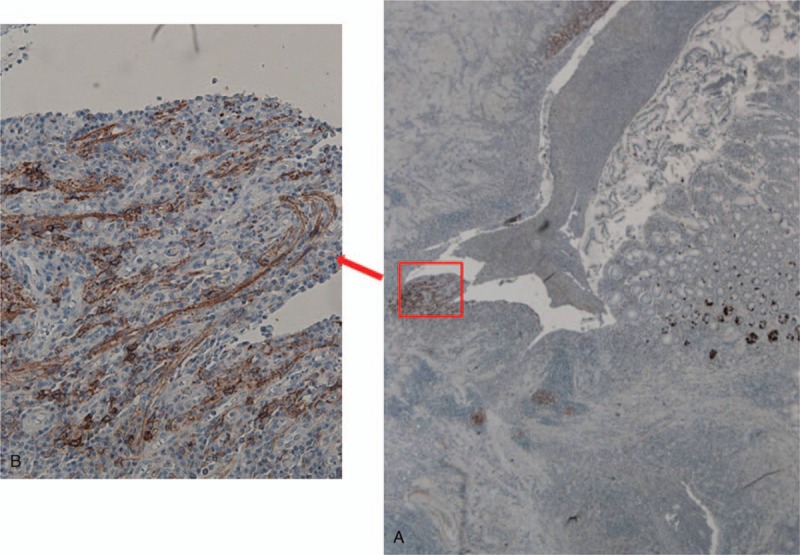
SSTR2A immunostaining (A–B): in nerve twigs of inflamed ulcerated mucosa deprived of glands, (20×–200×). SSTR2A = somatostatin receptor type 2A.

A 55-year-old woman with a medical history of CD, diagnosed in 1994 and surgically treated in 1995, was referred to our hospital for Crohn recurrence. Imaging and colonoscopy revealed a stenotic area in the neoterminal ileum, that was surgically treated. At 5 cm of preterminal ileum with stenosis and the anastomotic ileocolic block was removed. The post-surgical period was uneventfully. Histopathological examination showed an active Crohn small intestine with ulcerative and fissuring lesions associated with prominent fibrotic tissue proliferation, impinging on the muscolaris propria and also affecting the subserosal adipose tissue. Moreover, the stenosis was mainly sustained by mass-forming, prominent ganglioneuromatous hyperplasia at the submucosa and muscolaris layer, associated with marked follicular hyperplasia (Fig. 1D). The muscolaris mucosae was destroyed or showed marked hyperplasia and delamination and fusion with the muscolaris propria, resulting in obliteration of the submucosa. To better define the ENS morphological alterations, immunostaining was carried out with specific neurogangliar antibodies on different areas of small intestine and in non-involved wall of colon cancer specimens used as control. Following the study by Chen^[6]^ and Tertychnyi^[9]^, we immunostained for PD-L1 and SSTR2A to investigate its specific features in CD. Complete results and specific locations are listed in Table 1. Normally in non-inflamed gut and control cases, few nerve fibers are seen in the muscolaris mucosa, with very rare fine nerve twigs extending up into the lamina propria running parallel to the crypts (Fig. 1A), with a frequency of 1 every 3 to 4 crypts. Anti-synaptophysin and neurofilament antibodies highlighted large amounts of small, arborizing nerve fibers in the mucosa (Fig. 1B–C) and in bundles of nerve twigs lying in the delaminated muscolaris mucosae of our patient. In addition, in the mucosa we found a new-formed fibrillary network originating from the hypertrophic submucosal and myenteric plexuses, extending into the inflammation area at the subepithelial luminal mucosa site,^[1]^ and immunostained by PD-L1 and SSTR2A antibodies (Fig. 2). Only described on GIST-1 (Dog-1) showed a focal positivity in the myenteric and submucosal plexuses but not in the hyperplastic neuromatous lesion and ganglion cells. On the contrary, anti-PD-L1 antibody weakly stained normal tissue plexuses but heavily stained the hyperplastic lesion and its bundles intermingled with muscolaris mucosae (Fig. 2) and twigs, scattered among inflammatory cells, in the mucosa. Moreover, in our material SSTR2A was identified both in T-lymphocytes and in a nerve network in the mucosa under the luminal epithelium (Fig. 3). This antibody reaction, negative in the control cases, can help to discriminate IBD from other causes of colonic inflammation, facilitating the identification of neural twigs in the mucosa of CD patients and predictive of neuromatous hyperplastic lesions deep in the wall. After surgery the patient was semestrally followed: the clinical and endoscopic evaluation was normal.

**Table 1:**
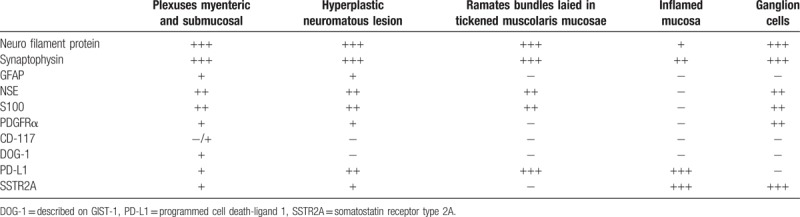
Immunohistochemical staining and its localization.

## 3 Discussion

An electron microscopy study reported that normal intestinal nerves are infrequently identifiable, unless they are proven neuromatous lesions.^[14]^ Recurrence of CD after gut resection is common and can be predicted by plexites. The main complications of CD, as in our case, are fibrotic strictures that lead to intestinal obstruction. Although it is well known that the diagnostic key to Crohn is a focal or patchy, discontinuous chronic pattern of inflammation (statement 22 of ECCO-ESP),^[18]^ many conditions, ranging from the undeclared use of local therapy to very early disease phases, as well as pediatric cases, can make differential diagnosis with UC a difficult task. In the initial biopsy diagnosis of Crohn, any other additional features^[19,20]^ must be searched for, and will result extremely useful in distinguishing Crohn from other IBD or recent described CEAS (chronic enteropathy associated with the SLCO2A1 gene).^[21]^ It has been suggested that the diagnosis of CD should be based on the presence of 2 major or at least 3 minor criteria, in the absence of granulomas. In daily histological diagnostic practice in our specialized gastrointestinal institute, great attention is paid to searching for ganglionites, regarded as one of the minor diagnostic criteria, together with lymphocytostasis, lymphangectasia, and thickening of muscolaris mucosae, that are sometimes detectable in routine biopsies. However, ganglioneuromatous changes, not present in mucosal biopsies and in conventionally stained specimens, remain difficult to visualize. Further prospective studies of the utility of PD-L1 and SSTR2A immunohistochemistry (IHC) in cases of focal chronic active inflammation raising the suspicion of CD, where the expected intense staining of numerous mucosal nerve twigs may help in the diagnostic definition, are warranted. We believe evaluation of the immunostaining of numerous high density fragmented fibers in subepithelial luminal portions of inflamed mucosa deprived of glands may also be useful. In particular, we highlight for the first time the detection of PD-L1 in plexuses and, with increasing intensity, in hyperplastic neuromatous lesions in Crohn disease, present in bundles of nerve twigs lying in the thickened muscolaris mucosae containing pain-sensing nerve fibers. The PD-L1/PD-1 pathway can regulate pain sensitivity via non-immune modulation processes such as neuronal modulation. It is increasingly appreciated that primary nociceptive neurons share similarities with immune cells and can both listen to and talk to immune cells.^[22–24]^ These results suggest that, given the high potency of PD-L1 in suppressing human nociceptors activities, local targeting of PD-L1/PD-1 signaling in sensory neurons may promote the development of novel analgesics.

Also mucosal twigs SSTR2A positivity can have a promising predictive role as a marker of underlying ganglioneuromatous lesions in Crohn because they are present only at the level of voluminous nodular submucosal trunks. Hence, the predictive value of subluminal SSTR2A positivity needs to be tested in different settings to evaluate its utility in clinical practice. Therefore, inflammation-related changes in ENS function likely involve neurodegeneration and neuroplasticity responsible for the main complication in CD, in some studies considered to be hamartoma.^[11–13]^ IHC for PD-L1 and SSTR2A supports the significance of new formed neural structures, long PD-L1 positive fibers parallel to the crypt and meshes consisting of neuronal fragments staining positive to SSTR2A, as exuberant repair processes secondary to chronic inflammation (Fig. 3). Also the differential intensity and location of nerves positive for DOG-1 and PD-L1 are consistent with this consideration. The high density of neural fibers in heavily inflamed mucosa around fistulo-stenotic lesions suggests that irritant proliferation stimuli are in progress and responsible for stenosis, due to huge nodular submucosal neural trunk formations that arise during recurrent disease lasting >10 years. In our case, stenosis as CD complication is due to hyperplastic neural lesions predicted by immunostainig of mucosal nerve twigs. In addition, specific nerve mediator components (SSTR2A) may be a target for immune attack, as a result of altered antigenicity following inflammation. Then the nervous system involved in IBD as a result of tissue injury may be a target for new therapies. It can be hypothesized that SSTR2A immunostaining would be useful to gauge the possible responsiveness, for some symptomatic traits of CD, to somatostatin analogs. However, the detection of PD-L1, prevalently on the hyperplastic trunk emphasizes the fact that further research is warranted to better define the potential of this observation for extending therapeutic opportunities. In conclusion, gut immunohistochemical assay of nervous bundles, allows new formed twigs to be visualized in mucosa, and their density and size evaluated. They can result predictive of deeper hyperplastic ENS changes, diagnostic of CD, and with a pathogenic role in stenosis.

## Acknowledgments

The authors are grateful to Antonio Cassano and Orazio Sabatelli for the professional technical support.

A. Cassano and O. Sabatelli give permission to be named.

## Author contributions

**Conceptualization:** Maria Lucia Caruso.

**Data curation:** Antonia Ignazzi, Sergio Coletta, Elisabetta Cavalcanti.

**Formal analysis:** Maria Lucia Caruso.

**Methodology:** Antonia Ignazzi, Sergio Coletta.

**Project administration:** Maria Lucia Caruso.

**Resources:** Maria Lucia Caruso.

**Supervision:** Maria Lucia Caruso.

**Validation:** Maria Lucia Caruso, Federica Di Pinto, Antonia Ignazzi, Sergio Coletta, Anna Maria Valentini, Elisabetta Cavalcanti, Francesco De Michele.

**Visualization:** Maria Lucia Caruso, Federica Di Pinto, Antonia Ignazzi, Sergio Coletta, Anna Maria Valentini, Elisabetta Cavalcanti, Francesco De Michele.

**Writing – original draft:** Federica Di Pinto, Antonia Ignazzi, Sergio Coletta, Anna Maria Valentini, Elisabetta Cavalcanti.

**Writing – review & editing:** Federica Di Pinto, Anna Maria Valentini, Elisabetta Cavalcanti, Francesco De Michele.

## References

[1] Geboes K Collins S Structural abnormalities of the nervous system in Crohn's disease and ulcerative colitis. Neurogastroenterol Motil 1998;10:189–202. 9659662 10.1046/j.1365-2982.1998.00102.x

[2] De Giorgio R Guerrini S Barbara G Inflammatory neuropathies of the enteric nervous system. Gastroenterology 2004;126:1872–83. 15188182 10.1053/j.gastro.2004.02.024

[3] Bondarenko VM Riabichenko EV Intestinal-brain axis. Neuronal and immune-inflammatory mechanisms of brain and intestine pathology. Zh Mikrobiol Epidemiol Immunobiol 2013;112–20. 23805681

[4] Cavalcanti E Armentano R Valentini AM Role of PD-L1 expression as a biomarker for GEP neuroendocrine neoplasm grading. Cell Death Dis 2017;8:e3004. 10.1038/cddis.2017.401 PMC5596583 28837143

[5] Valentini AM Di Pinto F Cariola F PD-L1 expression in colorectal cancer defines three subsets of tumor immune microenvironments. Oncotarget 2018;9:8584–96. 29492219 10.18632/oncotarget.24196 PMC5823560

[6] Chen G Kim YH Li H PD-L1 inhibits acute and chronic pain by suppressing nociceptive neuron activity via PD-1. Nat Neurosci 2017;20:917–26. 28530662 10.1038/nn.4571 PMC5831162

[7] Talme T Ivanoff J Hägglund M Somatostatin receptor (SSTR) expression and function in normal and leukaemic T-cells. Evidence for selective effects on adhesion to extracellular matrix components via SSTR2 and/or 3. Clin Exp Immunol 2001;125:71–9. 11472428 10.1046/j.1365-2249.2001.01577.x PMC1906108

[8] West NR Hegazy AN Owens BMJ Oncostatin M drives intestinal inflammation and predicts response to tumor necrosis factor-neutralizing therapy in patients with inflammatory bowel disease. Nat Med 2017;23:579–89. 28368383 10.1038/nm.4307 PMC5420447

[9] Tertychnyi AS Akhrieva KM Zayratyants OV Somatostatin receptor expression in the ileal mucosa of patients with inflammatory bowel disease. Arkh Patol 2016;78:19–24. 10.17116/patol201678119-24 26978232

[10] Steinhoff MM Kodner IJ DeSchryver-Kecskemeti K Axonal degeneration/necrosis: a possible ultrastructural marker for Crohn's disease. Mod Pathol 1988;1:182–7. 3237698

[11] Caruso ML Cavalcanti E De Michele F Small bowel capsule endoscopy revealing neuromuscular and vascular hamartoma of the jejunum: a case report. Medicine (Baltimore) 2018;97:e0196. 29642143 10.1097/MD.0000000000010196 PMC5908559

[12] Crothers J Zenali M Neuromuscular and vascular hamartoma of the small intestine: an exuberant reparative process secondary to chronic inflammation. Int J Surg Pathol 2015;23:673–6. 26275621 10.1177/1066896915600518

[13] Liu N Pan Y Li ZS Neuromuscular and vascular hamartoma: a rare entity or special phase of Crohn's disease. J Dig Dis 2015;16:52–4. 25223622 10.1111/1751-2980.12192

[14] Dvorak AM Silen W Differentiation between Crohn's disease and other inflammatory conditions by electron microscopy. Ann Surg 1985;201:53–63. 3966828 PMC1250618

[15] O’Morain C Bishop AE McGregor GP Vasoactive intestinal peptide concentrations and immunocytochemical studies in rectal biopsies from patients with inflammatory bowel disease. Gut 1984;25:57–61. 6360814 10.1136/gut.25.1.57 PMC1432236

[16] Bishop AE Polak JM Bryant MG Abnormalities of vasoactive intestinal polypeptide-containing nerves in Crohn's disease. Gastroenterology 1980;79 (5 pt 1):853–60. 7419008

[17] Sokol H Polin V Lavergne-Slove A Plexitis as a predictive factor of early postoperative clinical recurrence in Crohn's disease. Gut 2009;58:1218–25. 19625280 10.1136/gut.2009.177782

[18] Magro F Langner C Driessen A European consensus on the histopathology of inflammatory bowel disease. J Crohns Colitis 2013;7:827–51. 23870728 10.1016/j.crohns.2013.06.001

[19] Caruso ML Cavalcanti E Penna A Useful trick for discovering granuloma in gastric Crohn's disease. Inflamm Bowel Dis 2017;23:E42–3. 10.1097/MIB.0000000000001190 28816759

[20] Harbord M Eliakim R Bettenworth D Third European evidence-based consensus on diagnosis and management of ulcerative colitis. Part 2: current management. J Crohns Colitis 2017;11:769–84. 28513805 10.1093/ecco-jcc/jjx009

[21] Umeno J Esaki M Hirano A Clinical features of chronic enteropathy associated with SLCO2A1 gene: a new entity clinically distinct from Crohn's disease. J Gastroenterol 2018;53:907–15. 29313109 10.1007/s00535-017-1426-y PMC6061663

[22] Ji RR Chamessian A Zhang YQ Pain regulation by non-neuronal cells and inflammation. Science 2016;354:572–7. 27811267 10.1126/science.aaf8924 PMC5488328

[23] Talbot S Foster SL Woolf CJ Neuroimmunity: physiology and pathology. Annu Rev Immunol 2016;34:421–47. 26907213 10.1146/annurev-immunol-041015-055340

[24] McMahon SB La Russa F Bennett DL Crosstalk between the nociceptive and immune systems in host defence and disease. Nat Rev Neurosci 2015;16:389–402. 26087680 10.1038/nrn3946

